# Olfactory testing in children using objective tools: comparison of Sniffin’ Sticks and University of Pennsylvania Smell Identification Test (UPSIT)

**DOI:** 10.1186/s40463-015-0061-y

**Published:** 2015-03-01

**Authors:** Sarah C Hugh, Jennifer Siu, Thomas Hummel, Vito Forte, Paolo Campisi, Blake C Papsin, Evan J Propst

**Affiliations:** Department of Otolaryngology – Head & Neck Surgery, University of Toronto, Toronto, ON Canada; School of Medicine, Queen’s University, Kingston, ON Canada; Department of Otorhinolaryngology, Interdisciplinary Center Smell & Taste, Technische Universitat Dresden, Dresden, Germany; The Hospital for Sick Children, Toronto, ON Canada

**Keywords:** Children, Olfactory testing, Smell

## Abstract

**Background:**

Detection of olfactory dysfunction is important for fire and food safety. Clinical tests of olfaction have been developed for adults but their use in children has been limited because they were felt to be unreliable in children under six years of age. We therefore administered two olfactory tests to children and compared results across tests.

**Methods:**

Two olfactory tests (Sniffin’ Sticks and University of Pennsylvania Smell Identification Test (UPSIT)) were administered to 78 healthy children ages 3 to 12 years. Children were randomized to one of two groups: Group 1 performed the UPSIT first and Sniffin’ Sticks second, and Group 2 performed Sniffin’ Sticks first and UPSIT second.

**Results:**

All children were able to complete both olfactory tests. Performance on both tests was similar for children 5 and 6 years of age. There was an age-dependent increase in score on both tests (p < .01). Children performed better on the Sniffin’ Sticks than the UPSIT (65.3% versus 59.7%, p < .01). There was no difference in performance due to order of test presentation.

**Conclusions:**

The Sniffin’ Sticks and UPSIT olfactory tests can both be completed by children as young as 5 years of age. Performance on both tests increased with increasing age. Better performance on the Sniffin’ Sticks than the UPSIT may be due to a decreased number of test items, better ability to maintain attention, or decreased olfactory fatigue. The ability to reuse Sniffin’ Sticks on multiple children may make it more practical for clinical use.

## Background

Olfaction plays an important role in maintaining awareness of one’s surroundings through detection of pleasant and noxious odors and contributes to the perception of flavor. Structural pathology preventing odorants from binding to olfactory receptors or any lesion along the olfactory pathway from the olfactory epithelium to the olfactory cortex may affect a person’s ability to perceive odors. Olfactory impairment has been described in patients with congenital syndromes, head trauma, chronic rhinosinusitis, nasal masses, and neurodegenerative and autoimmune diseases. Various medications and smoking have also been implicated as causes of olfactory dysfunction [1]. Poor olfactory function has been associated with a decreased quality of life [2].

Approximately 19% of adults have some form of olfactory dysfunction (13% hyposmia, 6% anosmia) [3]. The prevalence of olfactory dysfunction in children is unknown. Unfortunately, diagnosing olfactory disorders based on history alone underestimates true prevalence rates in adults [4]. This underestimation is likely much greater in children. Since it is important for people with olfactory dysfunction to obtain counselling regarding fire safety and food inspection, proper diagnosis of this condition with objective testing is paramount.

There are a number of objective psychophysical olfactory tests commercially available for clinical use in adults, and normative data have been collected and thresholds determined for hyposmia and anosmia [5]. In general, various odors are presented to participants who are required to identify each odor from a defined list in a forced choice paradigm. The two most commonly employed tests in adults are the Sniffin’ Sticks (Burghart Messtechnik, Wedel, Germany) and the University of Pennsylvania Smell Identification Test (UPSIT) (Sensonics Inc., Haddon Heights, New Jersey, USA) [6,7]. Sniffin’ Sticks constitutes a 12-item test whereby odors are presented via reusable odor-dispensing pens. The UPSIT is a 40-item test whereby odors are presented on one-time-use scratch-and-sniff paper. Normative data for Sniffin’ Sticks, based on a cohort of 201 healthy children aged 6 to 11 years, has been published [8]. Normative data for combined age categories of 5 to 9 years and 10 to 14 years are available for UPSIT [9,10]. Similarly, in adults, normal ranges of scores for olfactory tests vary according to age [6,7,11]. There has been limited use of these tests in younger children. Previous authors have found olfactory testing to be difficult and unreliable in children less than six years of age, due to lack of motivation to complete the test or difficulty in understanding test instructions [12]. Testing in young children is further complicated by their lack of familiarity with test odors [13]. Olfactory test batteries have been created for children, however, they are more difficult to obtain and are not widely used [13,14]. To date, there have been no studies comparing Sniffin’ Sticks to the UPSIT in children.

The purpose of this study was to obtain data for normal healthy children ages 3 to 12 years on both the Sniffin’ Sticks and the UPSIT and to compare performance on the two tests. We hypothesized that children less than six years of age would be able to complete olfactory testing, that scores on both tests would increase with increasing age, that performance would be better using Sniffin’ Sticks than the UPSIT given that Sniffin’ Sticks contains fewer test items, and that performance would drop off over time due to physical and olfactory fatigue.

## Methods

This project was approved by The Hospital for Sick Children Ethics Review Board, which adheres to the “Tri-Council Policy Statement: Ethical Conduct for Research Involving humans.” Healthy children aged 3 to 12 years were recruited through an ambulatory tertiary care Pediatric Otolaryngology clinic from May to August, 2013. Exclusion criteria included the following: 1) syndromic patients including craniofacial anomalies and developmental delay; 2) nasal obstruction or sinus complaints such as allergy or nasal polyposis; 3) symptoms or signs of recent (within prior 4 weeks) respiratory tract infection such as congestion, rhinorrhea, fever, sore throat, acute otitis media or otitis media with effusion; 4) sleep disordered breathing; 5) prior upper aerodigestive tract surgery within preceding year (including tonsillectomy and/or adenoidectomy); 6) comorbidity such as cardiovascular, endocrine, autoimmune or pulmonary disease; 7) head trauma. The majority of participants were healthy siblings who accompanied their sibling to their appointment or patients referred for otologic complaints.

Prior to enrolment in the study, children were screened for bilateral nasal patency using a mirror to detect condensation from each nostril. Children were randomized, using a computerized random number generator, to one of two groups: Group 1 performed the UPSIT first and Sniffin’ Sticks second, and Group 2 performed Sniffin’ Sticks first and UPSIT second. Randomization was performed to control for attentional or olfactory fatigue. To control for differences in reading comprehension, multiple choice answers were provided in written format and read aloud to children by the test administrator. Participants were forced to choose an answer for every odor presented. Answers were recorded by one of two administrators (SCH, JS) and there was no time limit for completion of either test. Statistical analysis (paired samples *t*-test and linear regression) were performed using IBM SPSS Statistics Version 22.0 (IBM, Armonk, New York), with significance set at a p < .05. A sample size calculation using numbers from van Spronson (2013) (p value of .05, power of .80, clinically significant difference of 1.86 and standard deviation of 1.63) revealed that 8 participants were required per age group.

## Results

**Figure 1:**
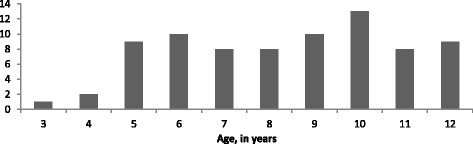
**Distribution of study participants by age in years.**

Seventy-eight children (43 male, 35 female) with a mean age of 8.4 ± 2.4 years (range 3 to 12 years) were included in this study (Table 1, Figure 1). Thirty-seven children were randomized to Group 1 and 41 children were randomized to Group 2. All participants completed both olfactory tests.

**Table 1:** **Demographic and olfactory testing data for study participants**

	**Group 1**	**Group 2**	**p-value**
	**(UPSIT performed first)**	**(Sniffin’ Sticks performed first)**	
**Age (SD)**	8.1 years (2.5)	8.6 years (2.4)	.34
**Sex**			
Male	18	25	
Female	19	16	
**Score**			
UPSIT	57.2%	61.3%	.32
Sniffin’ Sticks	60.1%	70.0%	.06

**Figure 2:**
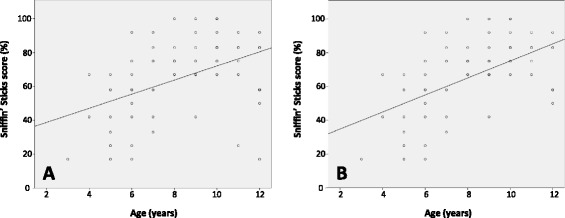
**Scatter plot of Sniffin’ Sticks scores, by age of participant, with line of best fit. A**, All participants included (R^2^ = 0.20; line of best fit: score = 4.17 x age + 30.4). **B**, Two outliers removed (R^2^ = 0.31; line of best fit: score = 5.1 x age + 24.5).

**Figure 3:**
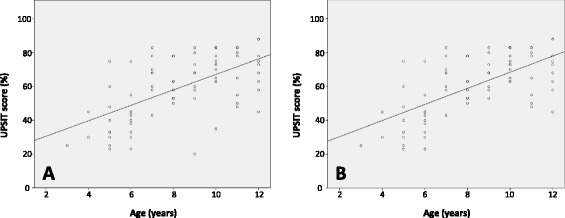
**Scatter plot of UPSIT scores, by age of participant, with line of best fit. A**, All participants included (R^2^ = 0.36; line of best fit: score = 4.57 x age + 21.4). **B**, Two outliers removed (R^2^ = 0.42; line of best fit: score = 4.76 x age + 20.9).

Children under the age of 6 years were able to complete both olfactory tests. Statistical analysis was not performed on children in age category of 3 years (N = 1) and 4 years (N = 2). Statistics were obtained from children as young as 5 years of age (N = 9) and there was no difference in Sniffin’ Sticks or UPSIT scores compared with scores from children 6 years of age (p = 0.11 and 0.80, respectively). Scores on Sniffin’ Sticks and UPSIT increased with increasing age in a linear fashion as demonstrated by regression analysis (performed between score and age, yielding R^2^ = 0.20 and 0.36, respectively, p < .01) (Figures 2 and 3). Removal of two outlying values for Sniffin’ Sticks scores (lowest Sniffin’ Sticks score for children of age 11 and 12 years, each lying more than two SD below the mean for their age category) resulted in an increase in R^2^ to 0.31. Effect size for analysis of variance (ANOVA) between age groups for Sniffin' Sticks scores was large (*n*^2^ = 0.47, outliers removed from analysis). Removal of two outlying values for UPSIT scores (lowest UPSIT score for children of age 9 and 10 years, each lying more than two SD below the mean for their age category) resulted in an increase in R^2^ to 0.42. Effect size for ANOVA between age groups for UPSIT scores was large (*n*^2^ = 0.55, outliers removed from analysis).

**Figure 4:**
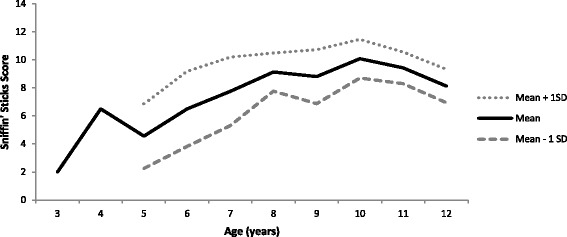
**Descriptive statistics of values for Sniffin’ Sticks, by age.**

**Figure 5:**
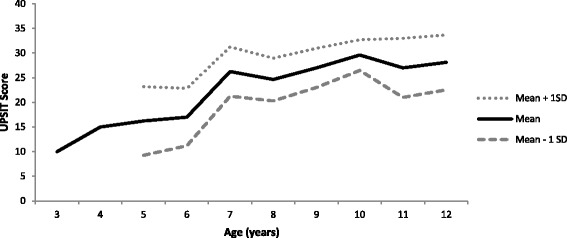
**Descriptive statistics ofvalues for UPSIT, by age.**

The overall mean score (SD) for Sniffin’ Sticks was 65.3% (22.6) and for the UPSIT was 59.7% (18.6). Paired *t*-test to compare the two means demonstrated a significant difference between participants’ scores (p < .01) with children performing better on Sniffin’ Sticks than on the UPSIT. There was no difference in Sniffin’ Sticks or UPSIT scores between Group 1 and Group 2 (Table 1). Descriptive statistics for values of Sniffin’ Sticks scores and UPSIT score by age (with outliers removed as described above) are shown in Figures 4 and 5, respectively.

## Discussion

The Sniffin’ Sticks and UPSIT olfactory tests were successfully administered to 78 children aged 3 to 12 years and data for this normal healthy population were obtained. All children, including those aged 3 to 5 years, were able to complete both tests. Children 5 years of age were capable of completing olfactory tests and did not score differently than children 6 years of age. Unfortunately, statistical analysis was precluded for age categories 3 and 4 years due to an insufficient number of participants. Contrary to previous findings, results suggest that testing may be extended to children 5 years of age.

Performance on both Sniffin’ Sticks and UPSIT increased with age. Effect size for ANOVA was large when analysed between age groups for scores on both tests. This is in keeping with previously demonstrated age-related increases in children’s performance on various olfactory tests [8,10,12,14,15]. However, we are unable to tell if this is due to development of the olfactory system over time, exposure to a wider variety of odors over time or simply due to broadening of the child’s lexicon. Thresholds for odor detection are similar in children and young adults, suggesting that performance on clinical olfactory tests depends not only on olfactory but also cognitive ability.

Overall mean scores for Sniffin’ Sticks were higher than on the UPSIT. This is in keeping with our hypothesis that children would perform better on the shorter 12-item Sniffin’ Sticks test than on the 40-item UPSIT. Better performance on the test with fewer items may have resulted from a faster administration time, greater ability to pay attention, or less olfactory fatigue. This may have practical implications whereby a shorter test battery may be more desirable in a busy clinical setting. However, the absolute difference between Sniffin’ Sticks and UPSIT scores should be interpreted with caution, as there is insufficient evidence to conclude that this difference is clinically significant. Further research in this area is warranted.

The order of test presentation was randomized to control for attentional or olfactory fatigue. Interestingly, there was no difference in Sniffin’ Sticks or UPSIT scores regardless of the order of test presentation. We conclude that differences in performance across tests were more likely due to inherent features of the tests themselves rather than the experimental condition.

A limitation of this study is the small sample size and limited number of participants under the age of 5 years. A larger sample size may elucidate the utility of these tests amongst younger children and perhaps even differences across sexes. Future studies comparing performance on these tests with other tests of olfactory function designed specifically for children are warranted. It would also be interesting to compare these perceptive tests of olfactory function to objective measures of olfaction.

## Conclusions

The Sniffin’ Sticks and UPSIT olfactory tests can both be completed by children as young as 5 years of age. Performance on both tests increased with increasing age. Children performed better on the Sniffin’ Sticks than on UPSIT, which may be due to a decreased number of test items, resulting in better ability to maintain attention or decreased olfactory fatigue. The ability to reuse Sniffin’ Sticks on multiple patients may make it more practical for clinical use.

## Abbreviations

SD: Standard deviation
UPSIT: University of Pennsylvania Smell Identification Test
ANOVA: Analysis of variance
